# The Allometry of Host-Pathogen Interactions

**DOI:** 10.1371/journal.pone.0001130

**Published:** 2007-11-07

**Authors:** Jessica M. Cable, Brian J. Enquist, Melanie E. Moses

**Affiliations:** 1 Department of Botany, University of Wyoming, Laramie, Wyoming, United States of America; 2 Department of Ecology and Evolutionary Biology, University of Arizona, Tucson, Arizona, United States of America; 3 Department of Computer Science, University of New Mexico, Albuquerque, New Mexico, United States of America; Columbia University, United States of America

## Abstract

**Background:**

Understanding the mechanisms that control rates of disease progression in humans and other species is an important area of research relevant to epidemiology and to translating studies in small laboratory animals to humans. Body size and metabolic rate influence a great number of biological rates and times. We hypothesize that body size and metabolic rate affect rates of pathogenesis, specifically the times between infection and first symptoms or death.

**Methods and Principal Findings:**

We conducted a literature search to find estimates of the time from infection to first symptoms (*t_S_*) and to death (*t_D_*) for five pathogens infecting a variety of bird and mammal hosts. A broad sampling of diseases (1 bacterial, 1 prion, 3 viruses) indicates that pathogenesis is controlled by the scaling of host metabolism. We find that the time for symptoms to appear is a constant fraction of time to death in all but one disease. Our findings also predict that many population-level attributes of disease dynamics are likely to be expressed as dimensionless quantities that are independent of host body size.

**Conclusions and Significance:**

Our results show that much variability in host pathogenesis can be described by simple power functions consistent with the scaling of host metabolic rate. Assessing how disease progression is controlled by geometric relationships will be important for future research. To our knowledge this is the first study to report the allometric scaling of host/pathogen interactions.

## Introduction

Most emerging infectious diseases that cause human epidemics (e.g. HIV, Influenza, West Nile Virus, Ebola) evolved in other animal hosts [1], [2]. However, little theory exists that enables the translation of our knowledge about pathogenesis, rates of evolution, vaccination strategies or epidemiology in these zoonotic diseases to their behavior in human hosts. One important observation is that the rate pathogens spread through populations appears to be influenced by the rate of spread through individual hosts [3], [4]. Therefore, understanding the time-course of pathogenesis in an individual, including the length of the latency and infection periods, could aid in parameterization of epidemiological models. A comparative approach to studying disease progression in different animal hosts may also elucidate how diseases affect human health.

Pathogenesis is a complex phenomenon that results from several aspects of host-disease interactions [5]. There are four main determinants of pathogenesis: (*i*) the interaction of the disease with the target tissue, (*ii*) the ability of the infection to cause cell death or cytopathology, (*iii*) the host immune response to infection, and (*iv*) immunopathology (e.g, T-cell and antibody responses). Although we know much about the physiological mechanisms for each of these host-disease interactions, there is still no easy answer for how pathogen infection ‘causes’ disease in a host [5]. Here we take a scaling approach to understand variation in the pace of pathogenesis. Specifically, what controls the scaling of pathogenesis times within and across diseases?

Here we focus on the role of host body size and metabolic rate in influencing the scaling of pathogenesis. There is a rich literature documenting how the body size of an animal influences its structure, function, and life history [6]–[11]. The overwhelming importance of body size has been eloquently summarized by George Bartholomew who stated, “It is only a slight overstatement to say that the most important attribute of an animal, both physiological and ecologically, is its size. Size constrains virtually every aspect of structure and function and strongly influences the nature of most inter- and intraspecific interactions. Body mass is the most widely used predictor of physiological rates.” [12]. However, little is known about the influence of host body size on pathogenesis in the context of the scaling of host-pathogen interactions. As we outline below, it is reasonable to expect that host body size and metabolic rate must constrain rates of pathogenesis.

### The Metabolic Scaling Theory (MST) for pathogenesis

Our hypothesis that host body size and ultimately host metabolic rate constrains rates of pathogenesis is based on recent theoretical developments for the scaling of biological rates and times [8], [10], [11]. Metabolic Scaling Theory (MST) predicts that physiological times and cellular metabolic rates are ultimately controlled by the scaling of the geometry of fractal-like vascular networks. This work secondarily hypothesizes that the scaling of physiological rates and times are governed by quarter-power scaling exponents. Specifically, quarter-power exponents in biology are the result of natural selection on vascular networks to minimize the scaling of internal transport times while maximizing the scaling of resource exchange surfaces (lung surface area, gut surface area etc). This work predicts a fractal-like vascular network design that, when scaled with the size of the organism in which it is contained, will lead to rates and times scaling as the 1/4 power of organism size. If rates of disease progression are ultimately constrained by network geometry and host metabolism, then pathogenesis will vary, or scale, with host body mass raised to a 1/4 power.

A number of studies have pointed to the fundamental importance of metabolism, or body size, in controlling the rates and timings of biological phenomena [9]–[11], [13]. This work has its roots in fundamental work by Kleiber [7]. Kleiber showed that whole organism metabolic rate, *B*, scaled allometrically (with an exponent less than 1) so that *B* = *B*_0_⋅*M* ^3/4^. Many subsequent studies supported this finding in a variety of taxa [6], [9], [14], [15], although others have questioned the generality of the 3/4 exponent [16], [17]. If *B*∝*M* ^3/4^ then the cellular or mass-specific metabolic rate, 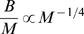 [[9 and refs therein,] [14]]. The theoretically predicted and empirically observed *M* ^−1/4^ decease in metabolism appears regulated by a similar *M* ^−1/4^ decrease in the amount of metabolic machinery. For example, the membrane surface area and number of mitochondria, concentration of ATP, number of cytochrome oxidase molecules, etc, all decrease with increasing host body size to the approximate −1/4 power [13], [18]–[20]. Since all other cellular rates are constrained by the metabolic rate of the cell, then the theory suggests that the rate of DNA synthesis, protein synthesis, immune response, and cellular turnover should also scale with *M* ^−1/4^, and biological times, *T*, should scale as the inverse of those rates, or as *M* ^1/4^ [9]. Cellular rates are relevant to pathogenesis because they control the rates at which pathogens enter a host, replicate, spread through the host body and cause disease.

Thus, according to the MST the pace of host-pathogen interactions (e.g. pathogenesis) is set by rate of host metabolism. The host metabolic rate influences pathogenesis by (*i*) constraining the rate of growth of pathogens that rely on host metabolic machinery (in much the same way as it limits the rate of growth of host [21]) as well as (*ii*) influencing the rate of the immune response of the host. In fact, cellular-mediated immunity appears to scale with body size and associated life history traits [22]. Thus, host metabolic rate influences the rate of pathogenesis since the ability of a pathogen to invade and replicate within a host may be driven by physiological rates and times of the host. Specifically, the times associated with pathogenesis are related to *M* by *t* = *c*⋅*M^b^* where *c* is a constant particular to the time of interest, and *b* = 1/4. Since mass specific metabolism (*B/M)*, scales as *M* ^−1/4^, then we expect rates of pathogenesis to scale with *M* ^−1/4^and times associated with pathogenesis to scale with M^1/4^. We assess these functional predictions with empirical data compiled from the literature. As far as we know, this is the first study to examine how pathogenesis varies as a function of host body size.

For a sampling of pathogens (one bacteria, three viruses, and one prion), we show how variation in host size, *M*, influences variation in the timing of pathogenesis. We focus on the time from inoculation to first symptoms (*t_S_*) and to death (*t_D_*). The MST predicts that both *t_S_* and *t_D_* will scale as the 1/4 power of mammalian body size, giving:
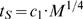
(1)and
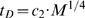
(2)The terms, *c_1_* and *c_2_*, in Eqs. 1 and 2 are scaling constants. The simplest model would have the values of *c_1_* and *c_2_* independent of *M*. Nevertheless, differences in their values reflect important interactions between host and pathogen and may be different for the pathogenesis of different diseases. We can combine Eqs. 1 and 2 to predict the relationship between *t_D_* and *t_S_*:
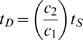
(3)The values of *c_1_* and *c_2_* likely reflect the timing of the host immune response and additional physiological responses to infection. These values may also be influenced by host body temperature, taxonomic group or other factors that alter host metabolism [10]. As *c_2_*>*c_1_*, 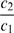 is the ratio between time to death and time to symptoms. Variability in this quotient between diseases would indicate proportional differences in the timing of between time until first symptom and time until death between diseases. However, similarity in this quotient between diseases would indicate generality in the proportional rates between diseases. Since our study is limited to homeotherms in similar taxonomic groups (mammals, and in the case of West Nile Virus, birds), within a particular pathogen, we expect *c_1_* and *c_2_* to be constant. If correct, then Eqs. 2–3 predict the timing of pathogenesis should be fundamentally set by host metabolism. However, for a given disease, the paces of various pathological events are predicted to be directly proportional, or isometric, to one another, so that *t_S_*∝*t_D_*. Further, the ratio of pathogenesis times should be independent of body mass, so that 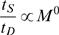.

### Alternative Hypotheses

We use MST to predict that *t_S_* and *t_D_* scale with M^1/4^. We contrast these predictions with the null hypothesis that *t_S_* and *t_D_* are independent of host body size (*M*). However, it is important to note alternative hypotheses that relate *t_S_* and *t_D_* to *M*. For example, symptoms or death may occur when some fraction of the number of cells in the organism has been infected. Since the number of cells is isometric with body mass [23], then *t_S_* and *t_D_* would be predicted to be linear with *M*. Alternatively the relationship between host mass and the timing of pathogenesis could be a geometric relationship controlled by body length or internal transport distances (i.e., in rabies or PRV, the distance the pathogen must travel from the site of infection to the brain). In simple Euclidean geometry, body length scales with body mass to the 1/3 power [e.g., 24]. The fractal network geometry of MST predicts that internal transport lengths show the same scaling as biological times; both scale with *M* ^1/4^. Here we specifically test the MST 1/4 power predictions, but we note which of the alternative predictions (*M* ^1^, and *M* ^1/3^) are consistent with the data.

## Results

**Figure 1:**
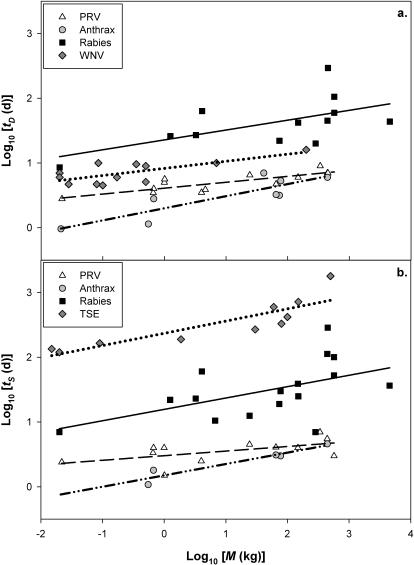
Time (days) from inoculation to (a) death and (b) 1^st^ symptom versus mammalian body mass for Pseudorabies virus (PRV), Anthrax, Rabies, West Nile Virus (WNV), and Transmissible Spongiform Encephalopathy (TSE).

**Figure 2:**
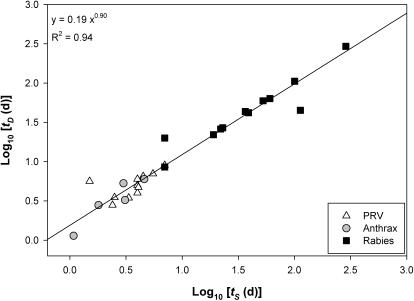
Time (days) from inoculation to death (*t_D_*) versus time from inoculation to 1^st^ symptom (*t_S_*) for Pseudorabies Virus (PRV), Anthrax, and Rabies for a large range of mammalian body sizes, plotted with the 1∶1 line.

We assessed the MST hypothesis by assembling data on the scaling of the timings of pathogenesis for five diseases. We collected data on *t_S_* and *t_D_* and *M* for a variety of pathogens infecting mammalian and bird species. Each of these diseases infect mammalian hosts that range in body size, *M*, by several orders of magnitude (e.g., Table 1). For the most part, empirical data support predictions made by the MST. In all five pathogens, there is a significant positive correlation between the timing of pathogenesis and *M* (Figure 1). For PRV, the scaling slope was positive, but was significantly lower than the predicted value of 1/4. For the remaining diseases, all slopes overlapped the predicted value of 1/4 (Table 1). The relationship between *t_S_* and *t_D_* had a slope close to 1 (Figure 2). Our results were not consistent with the alternative hypothesis that the timing of pathogenesis and M are isometric (slope of 1). However, the following diseases were consistent with the geometric hypothesis that pathogenesis times scale with mass to the 1/3: anthrax (*t_S_*, *t_D_*), rabies (*t_S_*, *t_D_*), TSE (*t_S_*) (Table 1).

**Table 1:** Slope and intercept (2.5%, 97.5% values), *R^2^*, *p* values, and body mass range (kg) for *t_S_* (time from inoculation to 1st symptom), *t_D_* (time from inoculation to death), and *t_S_* vs. *t_D_* for each disease*.

	Slope	Intercept	*R^2^*	*p*	Mass Range
***t_S_***					
Anthrax	**0.18** (0.11, 0.32)^ab^	0.17 (0, 0.33)^c^	0.90	0.013	0.55–442
PRV	0.12 (0.07, 0.21)^a^	0.43 (0.30, 0.55)^c^	0.33	0.051	0.022–442
Rabies	**0.33** (0.21, 0.54)^b^	0.93 (0.57, 1.29)	0.28	0.037	0.022–4545
TSE	**0.22** (0.15, 0.32)^ab^	2.35 (2.20, 2.52)	0.76	0.001	0.015–500
***t_D_***					
Anthrax	**0.22** (0.12, 0.39)^a^	0.29 (0.08, 0.51)^c^	0.62	0.021	0.022–442
PRV	0.11(0.08, 0.16)^b^	0.59 (0.53, 0.66)^d^	0.69	<0.001	0.022–450
Rabies	**0.26** (0.15, 0.44)^a^	1.2 (0.84, 1.5)^e^	0.35	0.041	0.022–4545
WNV	**0.17** (0.10, 0.28)^ab^	0.95 (0.84, 1.1)^e^	0.51	0.014	0.02–200
***t_S_*** ** vs ** ***t_D_***					
Anthrax	1.17 (0.65, 2.12)^a^	0.05 (−0.26, 0.37)^b^	0.88	0.018	0.55–442
PRV	0.83 (0.46, 1.48)^a^	0.23 (−0.06, 0.53)^b^	0.34	0.058	0.022–442
Rabies	0.82 (0.63, 1.06)^a^	0.34 (−0.01, 0.68)^b^	0.86	<0.001	0.022–4545

* PRV: Pseudorabies Virus, TSE: Transmissible Spongiform Encephalopathy, WNV: West Nile Virus

Significant *p* values (<0.05) denote slopes that differ from 0. Bolded slope values do not differ from 0.25. Slope and intercept values that differ among diseases (but within each time category) have different super-scripted letters. The intercept value for *t_S_* is *c_1_* and for *t_D_* is *c_2_* (Eqns 1, 2).

**Figure 3:**
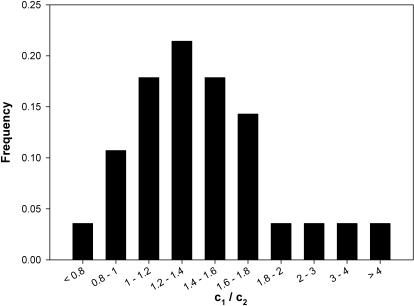
The frequency of 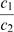 values for Pseudorabies Virus, Rabies, and Anthrax across a large range of mammalian body sizes.

There was variation in the scaling constants for each disease (Table 1), as exemplified in Figure 1 where the scaling slope was very similar but the intercepts (which gives *c_1_*) differed. The values of *c_1_* and *c_2_* ranged from 0.64 to 4.4. For example, for PRV, the time until death, *t_D_*, was 2.8 days for a 21 g mammal, whereas in Rabies the same size mammal was characterized by *t_D_* of 8.6 days. However, there was much less variation in the ratio of 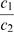 as shown in Figure 3, where 86% of the values fell between 0.8–1.8 (Figure 3).

## Discussion

Our results are generally consistent with the MST, where the timing of pathogenesis is controlled by host cellular metabolic rate. That is, the progression of disease to symptoms and to death slows as a function of *M* ^1/4^. Variation in *t_S_* and *t_D_* for each disease appears to scale with host body size with exponents consistent with the scaling of host metabolism. Observed relationships all scale with exponents very close and often indistinguishable from the predicted value of 1/4 (Figure 1).

As indexed by the fitted allometric intercepts, each disease differs in the relative timing of *t_S_* and *t_D_* (i.e. host-pathogen interactions differ in their value of *c_2_* and possibly *c_1_*). A plot of *t_S_* vs. *t_D_* across the diverse diseases studied reveals that the timing of pathogenesis for each disease, remarkably, falls on the same function that is approximately isometric (slope of 1) (Figure 2). Such invariance indicates that the allometric value of the ratio 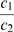 (see Eq. 3) is the same invariant quantity for each of the diseases studied here. We also provide a histogram of 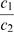 to show this ratio typically has a mean value of 1.6 (standard deviation 0.80) (Figure 3) and does not change systematically with *M*. This implies a relationship, general among these diseases, whereby the time to the first sign of infection is a constant proportion of the time to death–a constant that is conserved across each of the diseases studied here. The histogram of 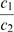 shows a long tail (Figure 3); perhaps these outliers are influenced by host immune response, medial care in humans, or specific host-pathogen interactions. Further investigation of pathogenesis in these mammals (cat, human, camelid, and elephant) may shed more light on mechanisms of allometric pathogenesis. It would also be interesting to understand how variation in evolutionary forces on these organisms affects host-pathogen interactions.

The scaling for PRV appears to not follow the predicted pattern of timing of pathogenesis as strongly as the other four diseases. PRV has a positive trend in the scaling relationship with significant slopes but they are more-shallow than predicted. It is unclear why PRV differs from the other diseases. Nevertheless, our model provides a baseline to begin to explore why PRV may deviate from the exact predictions of the MST. Explaining the causes of variation around the regression lines in Figures 1 is a natural, and we believe, fruitful next step to this analysis.

Our results also indicate that disease allometry across diverse populations may be characterized by invariant dimensionless quantities. Because mammalian life-span and population doubling time scale as *t_LS_* = *c*_3_⋅*M* ^1/4^ and *t_P_* = *c*_4_⋅*M* ^1/4^, respectively [9], where *c_3_* and *c_4_* are allometric constants with units of time, and if *t_D_* = *c*_2_⋅*M* ^1/4^, then the values for both 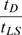 and 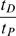 are equal to:
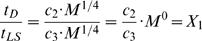
(4)

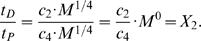
(5)Note, both *X_1_* and *X_2_* are dimensionless ratios invariant of mammalian body size. Thus, remarkably, across all mammals the fraction of adult lifespan or population cycle influenced by a given disease is an approximately constant value independent of mammalian body size.

We have shown that the scaling of times associated with pathogenesis is consistent with the scaling of host metabolic rate, supporting the MST. We have suggested that such scaling could result if pathogen growth and replication are directly limited by the cellular metabolic rates of the hosts. We are not aware of any other model(s) that would lead to functional relationships of *t_S_* and *t_D_* that are power-functions of body mass with exponents near 1/4. However, it is possible that the observed scaling could be an indirect result of metabolic rate. For example, host immune and other physiological responses to pathogens may cause the observed scaling, rather than the rate at which pathogens replicate, or the scaling may represent some combination of factors. It is also possible that pathogens may evolve latency periods in order to maximize their fitness given the population dynamics of the host. Evidence of this is seen in the evolution of *t_S_* in TSE. When laboratory mice are infected with TSE from larger animals (sheep or cows), *t_S_* is initially several times longer than after the infection has persisted in mouse populations for several generations. This effect is known as the ‘species barrier’ (Gardash'yan 1976, Nonno and Trevitt 2006; in supplementary material). Thus, when TSE is transmitted to a new, smaller, species, it evolves a faster *t_S_* after just a few generations.

We would like to note that an extensive survey of the veterinary and disease literature (see Supplementary Information) revealed only five diseases that allowed for sufficient body size variation and with enough reported values of pathogenesis times, and only three of those gave both time to symptoms and time to death. In future pathogenesis studies, we urge researchers to carefully report associated pathogenesis times as this will greatly increase the range of studies available for disease allometry, and greatly improve the ability to discriminate between MST and other hypotheses, such as the geometric hypothesis (scaling exponent of 1/3). While data were available for a large range of mammal body sizes (see Table 1), data were unavailable for animals at either extreme of the spectrum of body masses, such as shrews and whales. MST makes theoretical predictions for these animals. For example, in whales, experimental infection with disease would be very difficult. Our model, however, indicates that we would expect pathogenesis times for a blue whale to be about 1.5 orders of magnitude longer than for a 1 kg mammal.

Our results suggest that a comparative approach to pathogenesis is valuable, and that MST gives novel theoretical predictions for understanding the pace and progression of disease. While there is variation in the scaling relationships we show, there are clearly systematic and allometric (slopes less than 1) relationships between times of pathogenesis and body size. Our initial survey indicates that the observed scaling exponents are consistent with the scaling of host metabolic rate (MST). These results support the notion that the scaling of metabolism fundamentally constrains rates of pathogenesis. Furthermore, our results have important implications for epidemic models that often assume that the timing of and dynamics of pathogenesis is independent of host body size, metabolism, or pathogen transport times [3]. Our findings also suggest that a focus on the fundamental role of how the scaling of host metabolism influences the pace of pathogenesis could contribute to a mechanistic understanding of pathogenesis, and in turn, a foundation for predictive diagnostics, effective vaccination and therapy.

## Materials and Methods

### Empirical Data

Data were gathered from an extensive literature search, the references for which are supplied as supporting online material (Text S1). The data incorporated four measures of host-pathogen interactions including: *t_D_*, the time to death of the host from inoculation with the pathogen (as indexed by either the time when an individual animal died or the time at which 50% of the experimental population died from a lethal dose or LD_50_); *t_S_*, the time to first sign of infection from inoculation; and [*P*] the concentration of pathogen particles injected during the reported study (Table S1). Data stem from *in situ* experiments. Values of pathogenesis times were reported in the original citations listed in the supplementary information (Text S1). In general, each study reported the observed time of first infection, sign of infection, and death. Studies reported values for a single individual or for a population. When data were assembled from population observations the recorded times were average values.

Our literature survey revealed three diseases for which *t_D_*, and *t_S_* were measured for a sufficient number of mammalian hosts that span a sufficiently wide range of *M* to test the value of the scaling exponent. Note that both *t_D_* and *t_S_* were not reported for every animal (Table S1). The number of animals for each disease: Pseudorabies Virus or PRV (*Herpesvirus suis) n* = 16; Anthrax bacteria (*Bacillus anthracis) n = *11; Rabies virus *(Lyssavirus sp.) n* = 21, Transmissible Spongiform Encephalopathy (prion) *n* = 10, and West Nile Virus (flavivirus) *n* = 11. Each is extremely lethal in its host and exhibits characteristic symptoms. Mammalian hosts differed in *M* by approximately 5 orders of magnitude (ranging from mice to horses and bears, Table S1). We found data on *t_D_* (but not *t_S_*) for West Nile Virus (WNV) and *t_S_* (but not *t_D_*) Transmissible Spongiform Encephalopathy (TSE), diseases such as scrapie and mad cow disease that are caused by a prion pathogen.

### Analysis

We tested Eqs. 1 and 2 using reduced major axis (RMA) regression (analysis of covariance, ANCOVA) on log transformed data [SMATR, 25]. Each data point represents *t_S_* or *t_D_* and *M* for a particular pathogen in a particular host species. We treated each disease as a separate regression and estimated *c_1_* and *c_2_* for each disease. We also tested whether the ratio, 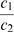, was constant across all pathogens by plotting log *c_1_* vs log *c_2_* and testing whether the slope of the RMA regression equals 1, and the group slope of PRV, Anthrax, and Rabies do not differ from 1 (*p* = 0.426) [26]. Since such methods do not necessarily indicate how much variation there is in that ratio [27] following Savage et al. (2006) [28] we further plot 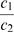 against M and provide a histogram of the values of 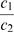 (Figure 3).

We did not incorporate phylogenetic corrections [29] in this analysis because it is not feasible for the limited number of animal hosts for which we have data. Nor did we attempt to look at the scaling of pathogenesis across growing individuals of the same species, again due to lack of data. If more data become available, we encourage such analysis in future tests of the MST for pathogenesis.

## Supporting Information

Table S1 The species, mass (kg), and time data collected from the literature. These data are from the literature listed in the supplementary material Text S1 and used in analyses across the five diseases; tD is the time to death from inoculation and tS is time to first symptom from inoculation (d). The five diseases are as follows: A = Anthrax, P = Pseudorabies Virus, R = Rabies, W = West Nile Virus, T = Transmissible Spongiform Encephalopathy. Where multiple masses are listed, the value used with each disease is noted with the letter of the disease (A, P, R, W, or T).
(0.11 MB DOC)

Text S1 Literature for disease data.
(0.05 MB DOC)
