# Supplementary material for: The Allometry of Host-Pathogen Interactions
Source: PLoS One. 2007 Nov 7;2(11):e1130. doi: 10.1371/journal.pone.0001130 (PMC2042517; doi:10.1371/journal.pone.0001130)
Supplement: Table S1 — The species, mass (kg), and time data collected from the literature. These data are from the literature listed in the supplementary material Text S1 and used in analyses across the five diseases; tD is the time to death from inoculation and tS is time to first symptom from inoculation (d). The five diseases are as follows: A = Anthrax, P = Pseudorabies Virus, R = Rabies, W = West Nile Virus, T = Transmissible Spongiform Encephalopathy. Where multiple masses are listed, the value used with each disease is noted with the letter of the disease (A, P, R, W, or T). (0.11 MB DOC) [file pone.0001130.s001.doc]

Table S1. The species, mass (kg), and time data collected from the literature.

| **Species** | **Scientific Name** | **Mass** | ***tD*** | | | | ***tS*** | | | |
| --- | --- | --- | --- | --- | --- | --- | --- | --- | --- | --- |
|  |  |  | **A** | **P** | **R** | **W** | **A** | **P** | **R** | **T** |
| American Crow | Corvus brachyrhynchos | 0.5 |  |  |  | 5.1 |  |  |  |  |
| Arctic/ Blue Fox | *Alopex lagopus* | 4 |  | 3.5 |  |  |  | 2.5 |  |  |
| Bank Vole | [*Myodes glareolus*](http://animaldiversity.ummz.umich.edu/site/accounts/information/Myodes_glareolus.html) | 0.02 |  |  |  |  |  |  |  | 120 |
| Black Bear | *Ursus americanus* | 150 |  |  |  |  |  |  | 25 |  |
| Black-billed Magpie | *Pica hudsonia* | 0.17 |  |  |  | 6 |  |  |  |  |
| Blue-Jay | *Cyanocitta cristata* | 0.08 |  |  |  | 4.7 |  |  |  |  |
| Camelids (lamiods) | *Lama spp.* | 568.2 |  |  | 59.5 |  |  |  | 52.5 |  |
| Cat | *Felis spp.* | 1.012 |  | 5.63 |  |  |  | 1.5 |  |  |
| Cheviot Sheep | *Bos spp.* | 80 |  |  |  |  |  |  |  | 330 |
| Common Grackle | *Ovis spp.* | 0.1 |  |  |  | 4.5 |  |  |  |  |
| Cow | *Quiscalus quiscula* | 442 (A, P, R), 500 (T) | 6 | 7 | 45 |  | 4.6 | 5.5 | 113 | 1800 |
| Dog | *Canis familiaris* | 24.3 |  | 6.5 |  |  |  | 4.5 | 12.5 |  |
| Drome-dary | *Camelus dromedarius* | 568.2 |  |  | 105.5 |  |  |  | 100.5 |  |
| Elephant - Asian | *Elephas maximus* | 4545.5 |  |  | 43.5 |  |  |  | 36.5 |  |
| Elk, Deer | *Cervus spp.* | 150 |  |  |  |  |  |  |  | 720 |
| Ferret | *Mustela nigripes* | 1 |  | 5 |  |  |  | 4 |  |  |
| Fish Crow | *Corvus ossifragus* | 0.28 |  |  |  | 9.6 |  |  |  |  |
| Goat -domestic | *Capra hircus* | 30 (T), 73.6 (A) | 3.17 |  | 22 |  |  |  | 19 | 270 |
| Gray Fox | *Urocyon cinereoargenteneus* | 4.09 |  |  | 63.5 |  |  |  | 60.5 |  |
| Guinea Pig | *Cavia porcellus* | 0.55 | 1.14 |  |  |  | 1.08 |  |  |  |
| Hamster | [*Mesocricetus auratus*](http://animaldiversity.ummz.umich.edu/site/accounts/information/Mesocricetus_auratus.html) | 0.085 (W), 0.09 (T) |  |  |  | 10 |  |  |  | 165 |
| Horse | *Equus caballus* | 200 (W), 450 (R, P) |  | 7 | 293.5 |  |  |  | 287 |  |
| House Finch | *Carpodacus mexicanus* | 0.02 |  |  |  | 7 |  |  |  |  |
| House Sparrow | *Passer domesticus* | 0.028 |  |  |  | 4.7 |  |  |  |  |
| Human | *Homo sapiens* | 60 (T), 77.3 (A, R) | 5.31 |  |  |  | 3 |  | 30 | 600 |
| Kodiak Bear | *Ursus spp.* | 570 |  |  |  |  |  | 3 |  |  |
| Lion | *Panthera leo* | 284 |  |  | 20 |  |  |  | 7 |  |
| Mink | *Mustela vison* | 0.66 |  | 3.46 |  |  |  | 3.35 |  | 190 |
| Mouse | *Mus spp.* | 0.015 (T), 0.02 (R), 0.021 (A), 0.0218 (P) | 0.96 | 2.8 | 8.55 |  |  | 2.4 | 7 | 135 |
| Pony | *Equus spp.* | 337.5 |  | 9 |  |  |  | 7 |  |  |
| Rabbit | *Sylvilagus spp.* | 0.672 | 2.8 | 4 |  |  | 1.8 | 4 |  |  |
| Raccoon | *Procyon lotor* | 4.62 |  | 3.88 |  |  |  | 0 |  |  |
| Rhesus Macaque | *Macaca fascicularis* | 1 (W), 5.34 (A), 6.7 (R) | 7 |  |  | 10 |  |  | 10.5 |  |
| Ring-billed gull | *Larus delawarensis* | 0.5 |  |  |  | 9 |  |  |  |  |
| Ringtail Possum | *Pseudocheirus spp.* | 3.25 |  |  | 27 |  |  |  | 23 |  |
| Sheep | *Ovis spp.* | 65 | 3.25 | 4.72 |  |  | 3.1 | 4.08 |  |  |
| Striped Skunk | *Mephitis mophitis* | 1.25 |  |  | 26 |  |  |  | 22 |  |
| Suffolk Sheep | *Ovis spp.* | 100 |  |  |  |  |  |  |  | 420 |
| White Tailed Deer | *Odocoileus virginianus* | 147.7 |  | 6 | 42 |  |  | 4 | 39 |  |

These data are from the literature listed in the supplementary material Text S1 and used in analyses across the five diseases; *t­D* is the time to death from inoculation and *tS* is time to first symptom from inoculation (d). The five diseases are as follows: A = Anthrax, P = Pseudorabies Virus, R = Rabies, W = West Nile Virus, T = Transmissible Spongiform Encephalopathy. Where multiple masses are listed, the value used with each disease is noted with the letter of the disease (A, P, R, W, or T).
