# Supplementary material for: The Allometry of Host-Pathogen Interactions
Source: PLoS One. 2007 Nov 7;2(11):e1130. doi: 10.1371/journal.pone.0001130 (PMC2042517; doi:10.1371/journal.pone.0001130)
Supplement: Text S1 — Literature for disease data. (0.05 MB DOC) [file pone.0001130.s002.doc]

Text S1. Literature for disease data.

http://www.fsis.usda.gov/Fact_Sheets/Bovine_Spongiform_Encephalopathy_Mad_Cow_Disease/index.asp.

(1965) Slow, Latent, and Temperate Virus Infections; Gajdusek DC, Gibbs CJ, Alpers M, editors. Washington, D.C.: U.S. National Institute of Neurological Diseases and Blindness.

(2000) Centers for Disease Control and Prevention. Use of anthrax vaccine in the United States: recommendations of the Advisory Committee on Immunization Practices (ACIP). Morbidity and Mortality Weekly Report 49(RR-15): 1-4.

(2001) Veterinary Services. Anthrax. Animal and Plant Health Inspection Service, USDA.

(2002) Transmissible Mink Encephalopathy Veterinary Services: http://www.priondata.org/data/A_tme.html.

(2005) The Birds of North America Online: http://bna.birds.cornell.edu/BNA/.

Apelt HJ (1983) Aujeszkysche krankheit bei Hund und Katze: Ein beitrang aus der praxis. Tierarztliche Praxis 11: 223-228.

Arora BM (1994) Viral Diseases. Wildlife Diseases in India: Infectious and Parasitic Diseases of Mammals, Reptiles and Amphibians. India: Periodical Expert Book Agency. pp. 68-80.

Banfield BW, Yap GS, Knapp AC, Enquist LW (1998) A chicken embryo eye model for the analysis of alphaherpesvirus neuronal spread and virulence. J Virol 72(6): 4580-4588.

Banks M, Torraca LSM, Greenwood AG, Taylor DC (1999) Aujeszky's disease in captive bears. Vet Rec 145(13): 362-365.

Bapu SR (1936-1937) A short note on elephants and a few of their common diseases. Indian Veterinary Journal 15: 36+43.

Beall FA, Dalldorf FG (1966) Pathogenesis of Lethal Effect of Anthrax Toxin in Rat. J Infect Dis 116(3): 377-&.

Belay ED, Maddox RA, Williams ES, Miller MW, Gambetti P et al. (2004) Chronic wasting disease and potential transmission to humans. Emerg Infect Dis 10(6): 977-984.

Belschner HG (1967) Rabies. Cattle Diseases. Sydney: Angus and Robertson. pp. 42-45.

Bitsch V, Knox B (1971) Pseudorabies in Carnivores in Demark .2. Blue Fox (Alopex-Lagopus). Acta Vet Scand 12(2): 285-&.

Bouzamondo E, Ladogana A, Tsiang H (1993) Alteration of Potassium-Evoked 5-Ht Release from Virus-Infected Rat Cortical Synaptosomes. Neuroreport 4(5): 555-558.

Brideau AD, Card JP, Enquist LW (2000) Role of pseudorabies virus Us9, a type II membrane protein, in infection of tissue culture cells and the rat nervous system. J Virol 74(2): 834-845.

Brossier F, Weber-Levy M, Mock M, Sirard JC (2000) Role of toxin functional domains in anthrax pathogenesis. Infect Immun 68(4): 1781-1786.

Bunning ML, Bowen RA, Cropp CB, Sullivan KG, Davis BS et al. (2002) Experimental infection of horses with West Nile virus. Emerg Infect Dis 8(4): 380-386.

Cantile C, Di Guardo G, Eleni C, Arispici M (2000) Clinical and neuropathological features of West Nile virus equine encephalomyelitis in Italy. Equine Vet J 32(1): 31-35.

Ceccaldi PE, Fillion MP, Ermine A, Tsiang H, Fillion G (1993) Rabies Virus Selectively Alters 5-Ht(1) Receptor Subtypes in Rat-Brain. Eur J Pharm-Molec Ph 245(2): 129-138.

Center NCD: National Oceanic and Atmospheric Association.

Choudhury B (1965) Incidence of anthrax in rhinoceros. Indian Veterinary Journal 42: 75-77.

Christodoulou T, Tsiroyiannis E, Papadopoulous O, Tsangaris T (1970) An Outbreak of Aujeszkys Disease in Minks. Cornell Vet 60(1): 65-&.

Constantine DG (1966) Transmission Experiments with Bat Rabies Isolates - Reaction of Certain Carnivora Opossum and Bats to Intramuscular Inoculations of Rabies Virus Isolated from Free-Tailed Bats. Am J Vet Res 27(116): 16-&.

Dietz O, Wiesner E (1984) Rabies. Diseases of the Horse: A Handbook for Science and Practice. Basel: Karger. pp. 278-282.

Dow C, Mcferran JB (1964) Experimental Aujeszkys Disease in Sheep. Am J Vet Res 25(105): 461-&.

Dow C, Mcferran JB (1966) Experimental Studies on Aujeszkys Disease in Sheep. Brit Vet J 122(11): 464-&.

Eldadah AH, Nathanso.N, Sarsitis R (1967) Pathogenesis of West Nile Virus Encephalitis in Mice and Rats .I. Influence of Age and Species on Mortality and Infection. Am J Epidemiol 86(3): 765-&.

Ernest SKM (2003) Life history characteristics of placental nonvolant mammals. Ecology 84(12): 3402-3402.

Etessami R, Conzelmann KK, Fadai-Ghotbi B, Natelson B, Tsiang H et al. (2000) Spread and pathogenic characteristics of a G-deficient rabies virus recombinant: an in vitro and in vivo study. J Gen Virol 81: 2147-2153.

Ezzell JW, Abshire TG (1992) Serum Protease Cleavage of Bacillus-Anthracis Protective Antigen. J Gen Microbiol 138: 543-549.

Ezzell JW, Ivins BE, Leppla SH (1984) Immunoelectrophoretic Analysis, Toxicity, and Kinetics of Invitro Production of the Protective Antigen and Lethal Factor Components of Bacillus-Anthracis Toxin. Infect Immun 45(3): 761-767.

Feder HM, Nelson RS, Cartter ML, Sadre I (1998) Rabies prophylaxis following the feeding of a rabid pony. Clin Pediatr 37(8): 477-481.

Fellows PF, Linscott MK, Ivins BE, Pitt MLM, Rossi CA et al. (2001) Efficacy of a human anthrax vaccine in guinea pigs, rabbits, and rhesus macaques against challenge by Bacillus anthracis isolates of diverse geographical origin. Vaccine 19(23-24): 3241-3247.

Fowler K, McBride BW, Turnbull PCB, Baillie LWJ (1999) Immune correlates of protection against anthrax. J Appl Microbiol 87(2): 305-305.

Friedlander AM (1999) Clinical aspects, diagnosis and treatment of anthrax. J Appl Microbiol 87(2): 303-303.

Gardash'yan AM (1976) Adaptation of Agent of Scrapie to Hamsters. B Exp Biol Med+ 81(2): 213-215.

Gleason SC, Currier R, Quinlisk P (1999) Public health response to a potentially rabid bear cub - Iowa 1999. Morbidity and Mortality Weekly Report 48: 971-973.

Gleiser CA (1967) Pathology of Anthrax Infection in Animal Hosts. Fed Proc 26(5): 1518-&.

Goto H, Gorham JR, Hagen KW (1968) Clinical Observation of Experimental Pseudorabies in Mink and Ferrets. Jpn J Vet Sci 30(5): 257-&.

Goto H, Burger D, Gorham JR (1971) Quantitative Studies of Pseudorabies Virus in Mink, Ferrets, Rabbits and Mice. Jpn J Vet Sci 33(3): 145-&.

Greene CE, Hall HF, Dreesen DW, Chandler FW (1984) Rabies. In: Greene CE, editor. Clinical Microbiology and Infectious Diseases of the Cat and Dog. Philidelphia: W.B. Saunders Company. pp. 356-380.

Hagemoser WA, Kluge JP, Hill HT (1980) Studies on the Pathogenesis of Pseudorabies in Domestic Cats Following Oral Inoculation. Can J Comp Med 44(2): 192-202.

Hanna P (1998) Current Topics in Microbiology and Immunology, Anthrax pathogenesis and host response. Berlin: Springer-Verlag.

Hara M, Shimizu T, Nemoto S, Fukuyama M, Ikeda T et al. (1991) A Natural Case of Aujeszkys Disease in the Cat in Japan. J Vet Med Sci 53(5): 947-949.

Henderson JP, Graham DA, Stewart D (1995) An Outbreak of Aujeszkys-Disease in Sheep in Northern-Ireland. Vet Rec 136(22): 555-557.

Husak PJ, Kuo T, Enquist LW (2000) Pseudorabies virus membrane proteins gI and gE facilitate anterograde spread of infection in projection-specific neurons in the rat. J Virol 74(23): 10975-10983.

Jackson AC (1999) Apoptosis in experimental rabies in bax-deficient mice. Acta Neuropathol 98(3): 288-294.

Jackson AC, Rossiter JP (1997) Apoptosis plays an important role in experimental rabies virus infection. J Virol 71(7): 5603-5607.

Kaplan AS (1974) The Herpesviruses; Kaplan AS, editor. U.s.: Academic Press. 739 p.

Kimman TG, Vanoirschot JT (1986) Pathology of Aujeszkys Disease in Mink. Vet Pathol 23(3): 303-309.

Kimman TG, Binkhorst GJ, Vandeningh TSGAM, Pol JMA, Gielkens ALJ et al. (1991) Aujeszkys Disease in Horses Fulfills Kochs Postulates. Vet Rec 128(5): 103-106.

Komar N, Langevin S, Hinten S, Nemeth N, Edwards E et al. (2003) Experimental infection of north American birds with the New York 1999 strain of West Nile virus. Emerg Infect Dis 9(3): 311-322.

Kornegay JN (1991) Multiple neurologic deficits. Inflammatory diseases. Problems in Veterinary Medicine 3(3): 426-436.

Lodmell DL, Ray NB, Parnell MJ, Ewalt LC, Hanlon CA et al. (1998) DNA immunization protects nonhuman primates against rabies virus. Nat Med 4(8): 949-952.

McLean RG, Ubico SR, Bourne D, Komar N (2002) West Nile virus in livestock and wildlife. Curr Top Microbiol 267: 271-308.

Merchant SR, Taboada J (1995) Systemic-Diseases with Cutaneous Manifestations. Vet Clin N Am-Small 25(4): 945-959.

Mocsari E, Szolnoki J, Glavits R, Zsak L (1989) Horizontal Transmission of Aujeszkys Disease Virus from Sheep to Pigs. Vet Microbiol 19(3): 245-252.

Mocsari E, Toth C, Meder M, Saghy E, Glavits R (1987) Aujeszkys Disease of Sheep - Experimental Studies on the Excretion and Horizontal Transmission of the Virus. Vet Microbiol 13(4): 353-359.

Mustafa AHM (1984) Isolation of Anthrax Bacillus from an Elephant in Bangladesh. Vet Rec 114(24): 590-590.

Nonno R, Di Bari MA, Cardone F, Vaccari G, Fazzi P et al. (2006) Efficient transmission and characterization of Creutzfeldt-Jakob disease strains in bank voles. Plos Pathog 2(2): 112-120.

Platt KB, Graham DL, Faaborg RA (1983) Pseudorabies - Experimental Studies in Raccoons with Different Virus-Strains. J Wildlife Dis 19(4): 297-301.

Quiroga MI, LopezPena M, Vazquez S, Nieto JM (1997) Distribution of Aujeszky's disease virus in experimentally infected mink (Mustela vison). Deut Tierarztl Woch 104(4): 147-150.

Rademacher G, Dirksen G, Straub OC (1991) Outbreaks of Aujeszkys Disease in Cattle Herds in Bavaria. Tierarztl Umschau 46(9): 513-520.

Ramachandran S, Natarajan M (1968) Tissue response to experimental anthrax of sheep and goats. Indian Veterinary Journal 45: 381-391.

Richards SH (1981) Miscellaneous Viral Diseases Diseases and Parasites of White Tailed Deer. In: Davidson WR, Hayes FA, Nettles VF, Kellogg FE, editors. Diseases and Parasites of White Tailed Deer. Tall Timbers Research Station, Tallahassee, FL: Miscellaneous Publication. pp. 108-125.

Ross JM (1957) The Pathogenesis of Anthrax Following the Administration of Spores by the Respiratory Route. J Pathol Bacteriol 73(2): 485-&.

Salman MD (2003) Chronic wasting disease in deer and elk: Scientific facts and findings. J Vet Med Sci 65(7): 761-768.

Scherer WF (1953) The Utilization of a Pure Strain of Mammalian Cells (Earle) for the Cultivation of Viruses Invitro .1. Multiplication of Pseudorabies and Herpes Simplex Viruses. Am J Pathol 29(1): 113-137.

Scherer WF, Syverton JT (1954) The Viral Range Invitro of a Malignant Human Epithelial Cell (Strain Hela, Gey) .1. Multiplication of Herpes Simplex, Pseudorabies, and Vaccinia Viruses. Am J Pathol 30(6): 1057-1073.

Scott PR (2002) Other Nervous Diseases. In: Martin WB, Aitken ID, editors. Diseases of Sheep. 3 ed. Oxford: Blackwell Science. pp. 228-240.

Sekhar PC, Singh RSJ, Sridhar MS, Bhaskar CJ, Rao YS (1990) Outbreak of Human Anthrax in Ramabhadrapuram Village of Chittoor District in Andhra-Pradesh. Indian J Med Res-A 91: 448-452.

Sen Gupta MR (1974) A preliminary report on diseases and parasites of zoo animals, birds, and reptiles. Indian Journal of Animal Health 13: 15-24.

Sikes RK (1962) Pathogenesis of Rabies in Wildlife .1. Comparative Effect of Varying Doses of Rabies Virus Inoculated into Foxes and Skunks. Am J Vet Res 23(96): 1041-&.

Steele JH (1973) The zoonoses: an epidemiologist's viewpoint. In: Stefanini M, editor. Progress in Clinical Pathology. New York: Grune and Stratton. pp. 239-286.

Stein CD (1947) Some observations on the tenacity of Bacillus anthracis. . Veterinary Medicine.

Swarbrick O (1967) Three incidents of anthrax. Vet Rec 80: 84-85.

Takahashi H, Yoshikawa Y, Kai C, Yamanouchi K (1993) Mechanism of Pruritus and Peracute Death in Mice Induced by Pseudorabies Virus (Prv) Infection. J Vet Med Sci 55(6): 913-920.

Takahashi H, Kai C, Yoshikawa Y, Yamanouchi K (1995) Immunohistochemical Analysis of Pseudorabies Virus Spread through Neurons Innervating the Eyeball. Comp Immunol Microb 18(4): 275-281.

Tierkel ES, Sikes RK, Starr LE (1970) Rabies. In: Gibbons WS, Catcott EJ, Smithcors JF, editors. Bovine Medicine and Surgery and Herd Health Management. Illinois: American Veterinary Publications. pp. 65-73.

Tizard IR (1987) Veterinary Immunology: An Introduction. Philidelphia: W.B. Saunders Company. 482 p.

Trevitt CR, Singh PN (2003) Variant Creutzfeldt-Jakob disease: pathology, epidemiology, and public health implications. Am J Clin Nutr 78(3): 651S-656S.

Umoh JU, Blenden DC (1982) The Dissemination of Rabies Virus into Cranial Nerves and Other Tissues of Experimentally Infected Goats and Dogs and Naturally Infected Skunks. Int J Zoonoses 9(1): 1-11.

Vandeningh TSGAM, Binkhorst GJ, Kimman TG, Vreeswijk J, Pol JMA et al. (1990) Aujeszkys Disease in a Horse. J Vet Med B 37(7): 532-538.

Vandevelde M (1984) Pseudorabies. In: Greene CE, editor. Clinical Microbiology and Infectious Diseases of the Dog and Cat. Philadelphia: W.B. Saunders Company. pp. 992.

Wernery U, Kaadan OR (2002) Infectious Diseases of Camelids: Blackwell Science. 418 p.

Xiao SY, Guzman H, Zhang H, da Rosa APAT, Tesh RB (2001) West Nile Virus infection in the golden hamster (Mesocricetus auratus): A model for West Nile encephalitis. Emerg Infect Dis 7(4): 714-721.

Zaucha GM, Pitt MLM, Estep J, Ivins BE, Friedlander AM (1998) The pathology of experimental anthrax in rabbits exposed by inhalation and subcutaneous inoculation. Arch Pathol Lab Med 122(11): 982-992.
